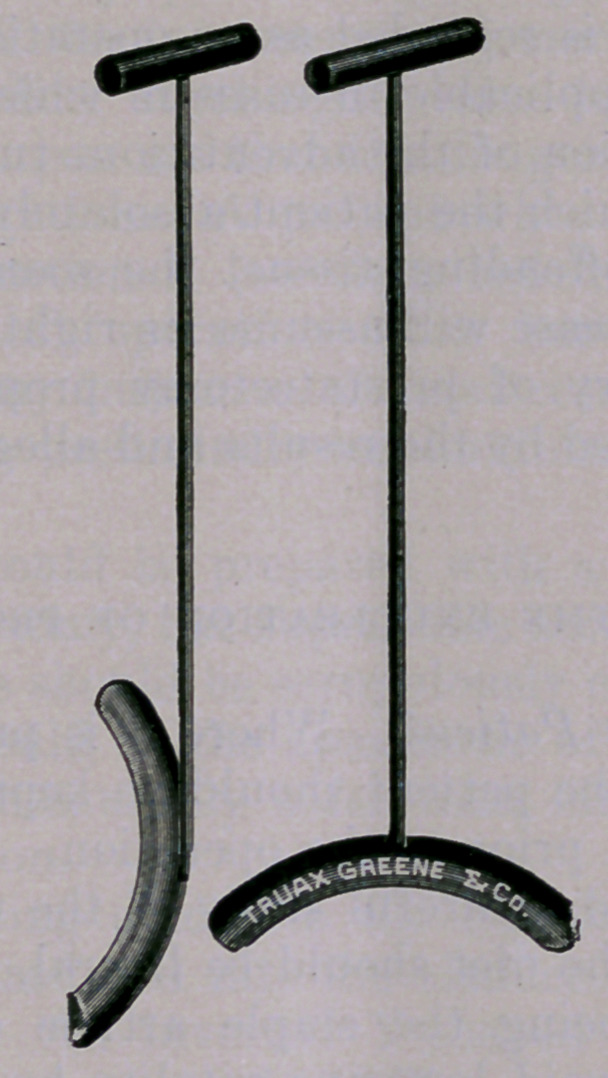# Prostatectomy; Its Indications and Technique*Read before the American Urological Association, New Orleans, May 8, 1903.

**Published:** 1903-10

**Authors:** G. Frank Lydston

**Affiliations:** Chicago, Ill.; Professor of the Surgical Diseases of the Genito-Urinary Organs and Syphilology in the Medical Department, Illinois State University; Surgeon to St. Mary’s Hospital


For Texas Medical Journal.

### Prostatectomy; Its Indications and Technique.*

BY G. FRANK LYDSTON, M. D., CHICAGO, ILL.

Professor of the Surgical Diseases of the Genito-Urinary Organs and Syphilology in the Medical Department, Illinois State University; Surgeon to St. Mary's Hospital.

The radical treatment of prostatic disease has at last assumed a position as a rational procedure, in harmony with advanced ideas of surgical pathalogy and surgical technique.

Prostatic surgery will be still more firmly established when the same rules have been applied to it as to other regions of the body considered amenable to operative interference. Time was when the

*Read before the American Urological Association, New Orleans, May 8, 1903.

operation of ovariotomy was performed only in extreme cases. The frightful mortality attendant upon it is a matter of history. All that is now necessary to prove the necessity of operation is to demonstrate the existence, or even the probable existence, of an ovarian cyst. This applies with equal force to the various operations for uterine fibroids, appendicitis, etc., and I hope will ere long apply to prostatectomy.

The past operative statistics of prostatic surgery are almost worthless, so far as establishing the value and safety of operations is concerned. Cases have been selected, not because they were suitable for operation, but usually because all other means have failed and serious complications have arisen.

In order that prostatic surgery should be given a fair opportunity to display its value, the most favorable time for operation should be selected. This is obviously the period before the patient is extremely advanced in years, and, more important still, before serious septic and secondary changes in the bladder, ureters and kidneys have occurred. In order that prostatic surgery should be placed upon a firm basis, several conditions should be complied with.

1. There should be a selection of cases in which a broad dividing line is drawn between those in which serious complications exist, and those in which they are absent. An operation performed even in patients of advanced years, in whom sepsis and renal disease have not developed, is comparatively safe. Prostatectomy compares very favorably with many operations which are considered less formidable, always providing it is performed at an early period in the development of the prostatic overgrowth. An operation upon the prostate in the presence of normal urine is, at least in the case of the perineal method, less formidable than many operations for stone, and when cases are properly selected the statistics of the surgery of the prostate will be of some value and the various radical operations will be shown to compare very favorably, as regards mortality, with other fields of operative work. In the collection of statistics, a broad dividing line should be drawn between the cases in which catheter life has not been established and the bladder and kidneys are sound, and those operated on after a more or less prolonged period of catheterization has elapsed and secondary bladder and renal changes have occurred. The former class is that upon which the statistics of the future will be based.

2. Both the profession at large and the laity should be impressed with certain fundamental facts regarding the prostate, namely, (a) the inevitable progress of prostatic overgrowth, when once it has commenced, in by far the majority of cases. The cases in which symptoms do not develop, and the patient consequently remains perfectly comfortable throughout his entire life, do not necessarily establish non-progression of prostatic overgrowth, but simply prove that some patients are exceptionally fortunate in that the mechanical conditions produced by it do not obstruct the urinary way. (b) The results of catheter life, during which infection almost inevitably occurs. The longevity of patients after the habitual use of the catheter has been once begun is, on the average, about five years, and those five years, in the majority of cases, hardly worth the living; in many instances by no means worth the living. The exceptions merely serve to prove the rule. (c) That an early operation, performed before the onset of bladder and renal complications, warrants a favorable prognosis, in by far the majority of cases.

3. The wisdom of an early diagnosis, to be followed by a radical operation, if the progress of the prostatic overgrowth is not speedily checked, is sufficiently obvious. The fatalism underlying the catheter habit, and the idea that all old men are doomed naturally to urinary disturbance, should be relegated to the valley of dead lumber. Both physician and layman should be taught the advisability of a careful supervision of the urinary apparatus of men at or above middle age. When symptoms are elicited, a careful examination should be made, and if enlargement of the prostate is found to exist, or develops later, a radical operation should be advised. There should be no compromise on the foregoing points, if mankind is ever to be freed from the misery produced by that *bête noir* of medicine—prostatic hypertrophy. The sooner the fallacious notion that all old men are legitimately entitled to misery during their declining years is exploded, the better for the profession, and, obviously, the better for the public at large.

In speaking thus emphatically I am by no means basing my position upon the recent operative furore in the direction of the prostate. Those of the profession who are familiar with my writings for many years past, and particularly the men whom I have had the honor of teaching, are well aware that my position has been a most uncompromising and radical one ever since the feasibility of the removal of prostatic overgrowths was demonstrated by Belfield, and McGill.

### INDICATIONS FOR OPERATIONS IN GENERAL.

I have long taken the radical ground that, other things being equal, the existence of prostatic overgrowth in any case warrants the consideration of a radical operation. The cases may be divided into

1. Cases at or moderately beyond middle life, in which the bladder and kidneys are yet normal.
2. The same class of cases, so far as age is concerned, complicated by septic vesical inflammation.
3. The same class of cases, complicated by a greater or less degree of secondary renal disturbance.
4. Patients of from fifty-five to sixty-five years of age, in whom no secondary conditions have yet occurred.
5. Cases of similar age, in which catheter life has been established for a greater or less length of time, and infection of the bladder has occurred.
6. Similar cases, in which the kidney is infected to a greater or less degree.

7. Patients above sixty-five years of age, with sound bladder and kidneys.

8. Patients above sixty-five years of age, in whom the bladder and kidneys are involved in secondary infection, to a greater or less degree.

9. Cases in very advanced life, in which the patient is suffering intensely, and more or less serious secondary involvement of the kidney and bladder is present.

Cases in comparatively young subjects, with sound bladder and kidneys, should be operated on, irrespective of the severity of the symptoms. The only exception is in cases in which the symptoms are slight, the prostatic enlargement of a simple hyperplastic nature, and the progress of the disease can be checked by such measures as dilation, general and sexual hygiene, and massage. Prostatic enlargement in such cases should be regarded, from an operative standpoint, as demanding the same radical treatment as any other surgical condition in which progressive development and serious secondary changes are almost inevitable. The prognosis in such cases is extremely favorable. The percentage of deaths should be almost *nil*. Cases in comparatively young subjects, in whom septic vesical inflammation exists, should always be operated, but, where possible, the bladder complication should be brought under control, and that viscus restored as nearly as possible to an aseptic and physiologic condition before operation. If this be done, the prognosis is almost, if not quite, as favorable as in the preceding class of cases. With proper drainage immediate operation offers a very good prospect of recovery.

In comparatively young subjects, with more or less secondary renal disturbance, with or without vesical sepsis which, of course, usually exists, operation offers, after proper preparatory treatment, a fair prospect of recovery, unless the seecondary renal disturbance is very marked. Even in these cases the results are sometimes surprising. I recall a case in which the specific gravity of the urine was only 1007 for many weeks; the patient greatly debilitated and anæmic from severe vesical hemorrhages; casts in the urine abundant; albumin in plenty, and the urea for many days about one-half of one per cent, but in which I performed an operation at the earnest solicitation of the patient. This case recovered completely, although the operation was a very formidable one. Uræmic symptoms were evident for several weeks.

The age of the patient is perhaps not so important as the relative degree of involvement of the kidneys; this, of course, within reasonable limits. Patients above sixty-five years of age, without secondary vesical or renal complications, offer a favorable prognosis in the majority of cases, the mortality, of course, increasing *pari passu* with the age of the subject.

Patients above seventy-five years of age, in whom vesical and prostatic symptoms have become manifest for the first time, should be offered in most cases the benefits of palliative treatment. Life expectancy is short in these individuals at best, and if the catheter and palliative treatment keep the patient perfectly comfortable, there is a question in my mind as to whether a radical operation should be seriously considered as a matter of routine. The expectancy of life should in each case enter largely into the consideration of the advisability of an operation. Cases of moderately advanced age, in which catheter life has been established for some time, should, as a rule, be operated on. If the kidney is seriously involved, operation should, in the majority of cases, be interdicted, save where all means of palliation fail to give the patient comparative comfort. When suffering is extreme and not amenable to palliation, operation is urgently necessary. Some patients would better chance dying on the operating-table than go on in their condition of distress. In such cases, however, palliative drainage should usually be the operation of election. A point which is not sufficiently emphasized is that in cases of this kind, to use an Irish bull, we ofttimes kill the patient by curing him, i. e., we remove the obstruction which is the cause of his suffering, but his life is destroyed by a lack of adaptability to the new conditions on the part of the bladder and kidneys. Ether nephritis and nephritis *ex vacuo* are very frequent. More frequent still is acute vesical, urethral and renal infection, superinduced by the relief of pressure, and constant circulatory and nutritive disturbance produced by the removal of the obstruction to the urinary outflow.

In a general way, patients with sound bladder and kidneys, from sixty-five to seventy-five years of age, offer a very favorable prognosis, providing the perineal operation is practicable. In very old patients with severe vesical and kidney infection, radical operatoins upon the prostate are usually contraindicated. The fact that this is the class of cases upon which the operative statistics of the prostate up to date have been largely based explains the discouragement which has been experienced in prostatic surgery.

### SELECTION OF OPERATION.

The various operations on the prostate have each their advocates, who claim that some particular method of operating is applicable to all cases. If this be true, then the prostate is an exception to all the rules of modern surgery.

The operative surgery of the prostate demands a differentiation of cases, and the adaptation of methods, not only to the various classes of cases, but to each individual case. The ideal operation is unquestionably the perineal method, where the conditions make it applicable. All cases are not susceptible to attack by the perineal route. When combined with suprapubic cystotomy, however, the perineal method enabels us to master most cases.

It is to be understood that "prostatectomy" of any variety is a misnomer. We simply shell out, so far as practicable, the tissue which comprises the greater portion of the bulk of the prostatic tumor. A certain amount of secondary inflammatory intra and extra-capsular tissue exists, which, with the capsule proper, is left behind by the operator. Once the tumors are enucleated, and the obstruction to the urinary way removed, nature takes care of the remaining adventitious tissue very nicely. Total prostatectomy is not practicable within the limits of safety. Furthermore, it is absolutely unnecessary. In cases in which a bar at the neck of the bladder exists, or there is a moderate median obstruction, without distinct tumor, the modified Bottini operation, via the perineum, or the direct use of the galvano-cautery through a fenestrated endoscope, is often effective, and is a rational method of procedure. Simple division of the bar—median prostatectomy—often gives perfectly satisfactory results. I have operated in a number of instances on the principle of the Abbe string saw, with advantage. A long, stiff, eyed probe is armed with a stout piece of silk, in which a number of knots have been tied. The probe is passed into and beneath the obstruction, at the neck of the bladder, and drawn out of the perineal wound by the finger, thus causing the ligature to traverse the tissue which it is desirable to divide. A slow sawing motion with the ligature, care being taken to protect the tissues of the perineum, soon divides the obstruction. Where the obstruction is not large, this method is often an excellent one, as the danger of hemorrhage is by it reduced to a minimum. Prolonged drainage with a rigid tube and the introduction of sounds during convalescence are usually necessary.

In cases in which palliative operation only is to be considered, they may be operated and the bladder drained, either from above or below. Great difficulty has been experienced, it is true, in maintaining a permanent suprapubic fistula which will permit the patient to keep clean and dry. If the opening in the bladder is made very small, dilatation of a small puncture rather than a large incision being relied upon, the puncture being made relatively close to the vesical neck, the fistula will usually be under control after healing is completed.

I have several cases under observation at present, in which the patients are so comfortable with a suprapubic fistula that they simply will not listen to the slightest suggestion of a radical operation upon the prostate. In one instance the old gentleman empties his bladder without any great difficulty at stated intervals, by the use of a glass funnel, which he applies over the fistulous opening.

In many cases the suprapubic, or the combined suprapubic and perineal sections are necessary. My rule is to remove the growth through the perineum, if practicable. In large, pedunculated growths, which sometimes practically fill the bladder; also in short, deep, fat perinei, it is sometimes with extreme difficulty that even the attachments of the tumors can be reached, much less their fundi.

Under these circumstances the combined or suprapubic operation is often imperative. In some of these cases hemorrhage is very serious, and suprapubic section, with packing of the bladder, is absolutely necessary. Where the prostratic tumor is large, and brought down with difficulty, or the perineum deep and short, I have not hitherto hesitated to resect the coccyx. This complicates matters very little, and practically does not add to the dangers of the operation, and adds surprisingly to the facility of reaching the pathologic tissue.

An instrument which I have used in a number of instances with satisfaction is presented herewith. (Figure.) It is introduced into a median incision at the apex of the prostate, the urethra being opened as a preliminary to the removal of the organ. The instrument is introduced closed, and is then opened with the finger. The vesical end of the instrument is patterned after the ordinary urethral sound, both ends being rounded. The convex border of the instrument rests upon the interior of the vesical neck, "perineumward." Considerable traction upon the prostate may be exerted with this instrument with perfect safety, and the extent to which the organ can be drawn down into the perineum is surprising to the uninitiated.

The sooner the profession is brought to realize the fact that no single operation can be successfully used in a routine manner in obstructive prostatic disease, the better for surgical science. We are justified in being suspicious of operators who report successful results from the routine application of any operative procedure. The various mutilating operations upon the testes and cord were boomed in the near past quite as enthustiastically as the Bottini operation has been of recent years. The history of the rise and fall of castration in prostatic disease is only too familiar. The conservatism of those who have hesitated to receive the Bottini operation as a routine procedure has been justified by the past history of prostatic surgery, and its wisdom is being daily confirmed by the experience of the profession at large.

The modified,—i. e., perineal,—Bottini has its limitations, and, in my opinion, they are very narrow. However modified, the Bottini method is, to my mind, a compromise with prostatic pathoolgy and surgery. While it may be useful in certain cases, it is demanded only because rational surgery is inapplicable, chiefly because of delay on the part of the patient in submitting to operation, or because of certain rare anatomo-pathologic conditions, which contraindicate the rational procedure of complete extirpation of the offending tissue. The less the Bottini operation is talked of, and the sooner it is regarded as an operation of necessity rather than election, and applicable in cases in which rational surgery, i. e., complete extirpation of the adventitious tumors, is not practicable, or in cases in which the patient absolutely refuses to submit to extirpation of the offending tissue, the sooner the radical treatment of prostatic disease will assume its rightful place in operative surgery. The history of prostatectomy proper is being and will still further be marred by the results and alleged results of surgical routinism.

### TECHNIQUE OF THE ENUCLEATION OF PROSTATIC TUMORS.

*Preparation of the Patient.* Where it is practicable to do so, as is usually the case, the patient should be kept quietly in bed from three days to a week prior to the operation. The kidneys demand careful attention, and a careful study of the urine should be made from day to day. The diet should be liberal, but red meats should be excluded, milk being the staple article of diet. Great care should be taken to avoid lowering vitality by a too restricted diet. This is a mistake that is very often made, and the patient goes to the operating-table with a resistancy lowered, rather than increased, by the preparatory regimen. Pure water should be given in abundance in most cases, although some discretion is necessary here. Urinary antiseptics are valuable. Urotropin, eucalyptus or boric acid are all of value, urotropin being, of course, the most efficacious of all. The bladder should be irrigated at stated intervals with mild antiseptic solutions. A mild solution of oxychlorine is often effective. Nitrate of silver, in weak solution, may be of service. If great irritation about the prostatic urethra exists, with frequent, painful and spasmodic micturition, anodynes should be given in sufficient quantity to control it, thus securing rest to the bladder. Anodynes act best when given by the rectum.

When the patient is placed upon the table for operation, the surgeon should understand that the correct method of operating will be governed largely by the character of the tumors. It is well to advise the patient, before operating, of the possible necessity of operating suprapubically. The exigencies of the case may, in exceptoinal instances, be indicated by the preliminary use of the cystoscope. In general, however, while this instrument adds to the accuracy of diagnosis, its use simply enhances the dangers of subsequent radical operations through the shock, traumatism and sepsis incidental to the exploration. Especially is this true where it is necessary to give an anæsthetic in order to explore the bladder with the cystoscope. There seems to me to be often very little sense in the refinements of cystoscopic diagnosis. In many cases of prostatic disease in which a radical operation is absolutely necessary the principal danger to the patient is from shock, acting reflexly upon the kidney, to which is added the further danger of anæsthesia. Why double the danger by preliminary diagnostic exploration, which modifies not at all the treatment, and merely gratifies the diagnostician without adding anything of value to the case? The impression forces itself upon me that the danger compounds very rapidly with every exploration. A patient who, having had a prolonged cystoscopic exploration a few days before, goes to the operating-table for a radical operation often has very little prospect of recovery. In many instances of fatal result after prostatic operations one or more preliminary explorations are responsible for the patient's death.

The patient should be prepared with a view to doing, not only perineal prostatectomy, but suprapubic section, if necessary. Shaving and antisepsis should be scrupulously carried out.

Chloroform should be the anæsthetic of election, for, as is well-known, it is immeasurably safer than ether in the class of cases under consideration.

The patient being placed in the lithotomy position, a medium-sized sound is introduced into the bladder and given into the hands of the assistant. An inverted Y-shaped incision is made in the perineum, the longer arm of the Y corresponding with the median raphe of the perineum, and the short arms traversing the region just in front of the anus. It is sometimes advantageous to make the lower incision curvilinear instead of Y-shaped. The length of the lower arms of the incision, or of the crescent, as the case may be, should be modified by the conformation of the perineum, which should be longer as the perineum is shorter, deeper and more fatty. The triangular flaps involved in the incisions should embrace all the tissues down to the muscular structure of the urethra. The triangular flaps having been dissected up cleanly, so as to expose the outlines of the urethra clearly, should be everted, and fastened to either buttock by a single strand of medium-sized silk traversing its free angle. Careful blunt dissection downwards along the urethra to the apex of the prostate must now be made, the rectum and the tissues in the ischio-rectal fossa being pulled down strongly by a retractor in the hands of an assistant. If sufficient room can not be obtained in this way, it can be increased greatly by resecting the coccyx, although this should be rarely necessary. As the dissection is being made, the assistant, using the sound as a lever, its convexity being directed perineumward, pries the prostate down into the wound as much as possible. Where the prostatic tumors are of moderate size and quite circumscribed, it is sometimes possible to enucleate them without opening the urethra. It is sometimes advisable to do this, unless the condition of the bladder be such as to positively demand drainage or intravesical work. Even here a retained catheter may be better than a perineal drain. Where a single prostatic tumor exists on one or the other side, an attempt should be made to enucleate it without opening the urethra, unless there be some positive indication for the latter procedure. A free incision in the capsule of the prostate is rarely necessary. I find that the best plan is to make a puncture with a pair of strong scissors. The puncture is enlarged by opening the blades of the scissors; the dilating finger of the operator does the rest. In some instances an incision of this kind should be made upon either side. Where opening the urethra seems advisable or unavoidable, the prostatic retractor shown herewith will be found exceedingly useful. (Figure.) An opening is made upon the staff at the apex of the prostate. Through this incision the prostatic urethra and neck of the bladder are thoroughly dilated with the finger so far as may be. The prostatic retractor is now inserted closed, and under the guidance of the index finger in the bladder, opened in such a manner that its convexity rests upon the lower segment of the bladder. The sound is, of course, removed before the neck of the bladder is dilated, and the retractor inserted. An intelligent assistant has now command of the situation. The slim wire shank of the instrument, while it is a guide, in a certain sense, to the enucleating finger, does not encroach by its bulk upon the field of operation. In some cases it is found impossible to pass a rigid staff into the bladder. Under such circumstances, one of two procedures may be resorted to: (1) A gum elastic catheter may be passed as a guide; (2) the urethra may be opened in the apex of the prostate upon a staff which can be readily passed down to this point. In enucelating the prostatic overgrowth, it should be remembered that in some instances damage may be done by over-zealousness in the attempt to remove all the adventitious tissue. No circumscribed overgrowth should be spared, but it should be understood that there is more or less diffuse tissue hyperplasia surrounding the tumors proper, and secondary to them, which it is both dangerous and unnecessary to remove, as it shrinks down readily when once the offending tissue has been extirpated. All distinct intumescences should be removed. Where enucleation is not practicable, *morcellment* with cutting forceps is required.

In cases in which distinctly circumscribed single or multiple tumors are not found, but where there is diffuse enlargement of one or both lobes, it is usually practicable to extirpate the entire mass by intracapsular enucleation. If a distinctly pedunculated or even plainly circumscribed median tumor exists, it may not be practicable to remove it extravesically. Under such circumstances it should be twisted off within the bladder, or enucleated through an incision upon its mucous surface.

The prostatic tumors having been removed, the bladder should be explored, and if stone be present it should be removed.

I desire to emphasize the fact at this juncture that in any instance in which the tumors are removed by incisions from the vesical side, the ideal perineal operation is distinctly debarred, and the operation reduced to the same plane, essentially, as that occupied by suprapubic cystotomy and intravesical extirpation of the prostatic overgrowths. Where the tumors are more or less polypoid in character, or if sessile, are situated very high up, their bulk being distinctly intravesical, it is occasionally necessary to combine suprapubic cystotomy with the perineal method, and remove the tumors under consideration from above.

The question of air versus fluid distention of the bladder during operation is largely a matter of taste. I have been in the habit of distending the bladder with either sterile water or weak antiseptic solutions, after thorough antiseptic irrigation. Drainage after the operation should be carried out by a large tube of some kind. Personally, I prefer a rigid tube, although in some instances I use a large rubber tube. The appliance used for drainage, like some other points in the technique, is largely a matter of individual taste. The duration of drainage should be governed by the condition of the bladder. I believe it is advantageous in some cases to dilate the neck of the bladder a few times before healing is complete.

---

In Praise of the Pituitary.—

The pituitary body and the infundibulum
Are each unto the other an inseparable chum.
For years the wise physicians have been puzzled by the twain—
The problem has bewildered every scientific brain.
The argument had risen to a stage intensely warm—
Some said it called for knives à la appendix vermiform.
But now the worth of science has been clearly shown again;
The pituitary body bosses all our oxygen.
The hot air we are handed is received as it may come,
And fitted for its journey through the infundibulum.
And thus we're benefited by the function pituitous,
And only get the portion that is nutriment to us.
When crafty politicians come to slap us on the back,
The pituitary body starts to taking up the slack;
It separates the nodules and the gases which are there
And hemoglobinizes that installment of hot air.
'Tis thus we see the blessings that unconsciously have come
Through the pituitary body and the infundibulum.
Give praise unto the surgeons whom the question could not tempt;
Who left the pituitary and the infundib. exempt;
Who yielded not, when nothing good of purpose they could see
About these minor organs, save an amputating fee;
And hail the gland so helpful and its ever-ready chum,
The pituitary body and the infundibulum! —*Chicago Tribune.*

## Figures and Tables

**Figure f1:**